# Translocation of molecular chaperones to the titin springs is common in skeletal myopathy patients and affects sarcomere function

**DOI:** 10.1186/s40478-017-0474-0

**Published:** 2017-09-15

**Authors:** Andreas Unger, Lisa Beckendorf, Pierre Böhme, Rudolf Kley, Marion von Frieling-Salewsky, Hanns Lochmüller, Rolf Schröder, Dieter O. Fürst, Matthias Vorgerd, Wolfgang A. Linke

**Affiliations:** 1 Department of Cardiovascular Physiology, Ruhr University Bochum, Bochum, Germany; 2 Institute for Genetics of Heart Diseases, University Hospital Muenster, Muenster, Germany; 3 Department of Neurology, Heimer Institute for Muscle Research, University Hospital Bergmannsheil, Ruhr University Bochum, Bochum, Germany; 4 Institute of Physiology II, University of Muenster, Robert-Koch-Str. 27b, 48149 Münster, Germany; 5 Institute of Genetic Medicine, Newcastle University, International Centre for Life, Central Parkway, Newcastle upon Tyne, UK; 6 Institute of Neuropathology and Department of Neurology, University Hospital Erlangen, Erlangen, Germany; 7 Institute for Cell Biology, Department of Molecular Cell Biology, University of Bonn, Bonn, Germany

**Keywords:** Myofibrillar myopathy, Muscular dystrophy, Muscle stiffness, Molecular chaperones, Immunoelectron microscopy

## Abstract

Myopathies encompass a wide variety of acquired and hereditary disorders. The pathomechanisms include structural and functional changes affecting, e.g., myofiber metabolism and contractile properties. In this study, we observed increased passive tension (PT) of skinned myofibers from patients with myofibrillar myopathy (MFM) caused by *FLNC* mutations (MFM-filaminopathy) and limb-girdle muscular dystrophy type-2A due to *CAPN3* mutations (LGMD2A), compared to healthy control myofibers. Because the giant protein titin determines myofiber PT, we measured its molecular size and the titin-to-myosin ratio, but found no differences between myopathies and controls. All-titin phosphorylation and site-specific phosphorylation in the PEVK region were reduced in myopathy, which would be predicted to lower PT. Electron microscopy revealed extensive ultrastructural changes in myofibers of various hereditary myopathies and also suggested massive binding of proteins to the sarcomeric I-band region, presumably heat shock proteins (HSPs), which can translocate to elastic titin under stress conditions. Correlative immunofluorescence and immunoelectron microscopy showed that two small HSPs (HSP27 and αB-crystallin) and the ATP-dependent chaperone HSP90 translocated to the titin springs in myopathy. The small HSPs, but not HSP90, were upregulated in myopathic versus control muscles. The titin-binding pattern of chaperones was regularly observed in Duchenne muscular dystrophy (DMD), LGMD2A, MFM-filaminopathy, MFM-myotilinopathy, titinopathy, and inclusion body myopathy due to mutations in valosin-containing protein, but not in acquired sporadic inclusion body myositis. The three HSPs also associated with elastic titin in mouse models of DMD and MFM-filaminopathy. Mechanical measurements on skinned human myofibers incubated with exogenous small HSPs suggested that the elevated PT seen in myopathy is caused, in part, by chaperone-binding to the titin springs. Whereas this interaction may be protective in that it prevents sarcomeric protein aggregation, it also has detrimental effects on sarcomere function. Thus, we identified a novel pathological phenomenon common to many hereditary muscle disorders, which involves sarcomeric alterations.

**Electronic supplementary material:**

The online version of this article (10.1186/s40478-017-0474-0) contains supplementary material, which is available to authorized users.

## Introduction

Hereditary myopathies are a clinically and genetically heterogeneous group of disorders with a variable age of onset, from congenital to late adulthood. The myopathies have been classified into subgroups based on the clinical distribution of muscle weakness (e.g. proximal vs. distal) and the inheritance pattern (e.g. autosomal dominant vs. recessive), before the genetic etiology was revealed [43]. Today, mutations in dozens of different genes are known to cause a myopathy [27], with Duchenne muscular dystrophy (DMD) due to mutations in dystrophin being the most frequent. A large subgroup encompasses the limb-girdle muscular dystrophies (LGMDs) caused by mutations in at least 30 different genes with autosomal dominant (LGMD1; 9 genes/loci) or autosomal recessive (LGMD2; 24 genes) inheritance [57]. One of the most frequent subtypes of LGMD is LGMD2A caused by homozygous or compound heterozygous mutations in the gene encoding the proteolytic enzyme calpain-3 (CAPN3), a known interactor of the giant sarcomere protein titin [16, 48]. Myofibrillar myopathies (MFMs), another group of hereditary muscle disorders, are characterized by histological features including focal disintegration of myofibrils and protein aggregation in myofibers. Known MFM disease genes encode proteins associated with the sarcomeric Z-disc, such as myotilin, desmin, and filamin-C [29]. For many LGMDs and MFMs, the molecular mechanisms underlying the respective disorder remain largely unresolved and specific or ameliorating therapies are not available.

In our previous approaches to this topic, we focused mainly on mechanisms of protein quality control, which we found to be pathologically altered in MFMs [30]. We deciphered distinct mutation-specific disease mechanisms in the human myopathies, such as protein misfolding and aggregation, toxic gain of function, and haploinsufficiency [2, 21]. Recently, we demonstrated that the small heat shock proteins (sHSPs), HSP27 (HSPB1) and αB-crystallin (HSPB5), which in healthy muscle cells localize to the Z-disc or cytosol, were translocated to the titin springs of the sarcomeric I-bands in LGMD2A myocytes [31]. Titin is established as being responsible for the elasticity and “passive” tension (PT) of the myocyte [39], but is also evolving as a protein critical for the active mechanical properties of the sarcomere [26, 36, 52]. The binding of HSPs to titin suggested a role for these molecular chaperones in the pathomechanism of myopathy subtypes, which are presenting with reduced contractile force generation and increased muscle stiffness.

HSPs are important components of protein quality control, as they affect protein folding and promote degradation, e.g. via the ubiquitin-proteasome system (UPS) or autophagy pathways [7, 56]. Members of the family of sHSPs assist in the folding and maintenance of the cytoplasmic proteome and are considered holdases rather than foldases [5, 42]. Interestingly, overexpression of sHSPs significantly reduces aberrant protein aggregation in cell and animal models of MFM [10, 53, 54, 59]. Moreover, chemical chaperones can impede pathological protein aggregation and improve muscle function [60]. We found that sHSPs stabilize folded immunoglobulin-like (Ig) domains of titin from the elastic I-band segment [9]. If these Ig modules unfold in response to sarcomere stretching [1, 52], the sHSPs may help protect them from aggregation [31]. The ATP-dependent chaperone HSP90 is known to assist in the assembly of the myosin filaments [55] and alter myosin motor function [47]. HSP90 also binds, if methylated by the methyltransferase Smyd2, to the N2A element of I-band titin (near the calpain-3-binding site) and exerts a protective effect on Z-disc/I-band structure [15, 58]. HSP90 is among the chaperones discussed as potential ameliorators of dystrophic muscle disease [8]. However, its relevance in LGMDs and MFMs has not been studied.

Considering the potential for chaperones to improve myocyte function in muscle disease, we initiated this study with the aim to better understand the relationship between titin and HSPs in human hereditary myopathies. We set out to determine which chaperones associate with titin in muscle biopsies from different myopathies, including LGMD2A and MFM-filaminopathy. We found that, of all HSPs studied, only HSP27, αB-crystallin and HSP90 were translocated from the cytosol or sarcomeric Z-disc in healthy human muscles to the titin springs in myopathy. We mapped the interaction sites by immunoelectron microscopy and measured the impact of endogenous HSP-binding to the sarcomeres on myofiber PT, in controls and myopathy patients. We also tested whether exogenous HSPs added to permeabilized human myofibers affect PT. By examining biopsy material from control subjects, 17 patients with different myopathies, and muscle from animal models of hereditary myopathies, we found that massive HSP-binding to titin is a common feature in dystrophic and MFM muscle disorders. We conclude that the translocation of HSPs to titin, while protecting the protein in the sarcomeres, could also impair titin-based myofiber elasticity, presumably contributing to increased muscle stiffness. These alterations represent a previously unrecognized pathophenomenon in hereditary myopathies.

## Methods

### Human muscle biopsies

We studied M. vastus lateralis and gastrocnemius biopsies from three healthy (CTRL) subjects with normal histopathological features and 17 diseased human subjects with various muscle disorders (see Table 1). The following hereditary myopathies were included (mutated gene and specific mutation(s) listed in parentheses): Among the group of muscular dystrophies, LGMD2A (*CAPN3*; p.Thr184Argfs & Trp130 > Cys, p.Thr184Argfs & p.Arg315Arg, p.Thr184Argfs & p.Gly329Arg) and Duchenne muscular dystrophy (*DMD*; Del. exon 2–18, Del. exon 3–11); among the group of myofibrillar myopathies, filaminopathy (*FLNC*; p.Val930_Thr/933del (2×)), desminopathy (*DES*; p.Arg350Pro), myotilinopathy (*MYOT*; p.Ser60Phe, p.Lys36Glu), and a titinopathy, hereditary myopathy with early respiratory failure (HMERF) (*TTN*; p.Cys30071Arg (2×)); hereditary inclusion body myopathy (inclusion body myopathy with Paget disease and frontotemporal dementia, IBMPFD) caused by valosin-containing protein mutation (*VCP*; p.Arg93Cys, p.Arg155His). At least two biopsy samples per disorder (in filaminopathy from two siblings) were analyzed, with the exception of desminopathy, from which only a single biopsy sample was available. Additionally, we included biopsies from three patients with acquired sporadic inclusion body myositis.

**Table 1:** Overview of human and mouse muscle samples studied and intracellular localization of major chaperones

			Intracellular localization of		
Gene	Disease/Mutation	Year of birth/Gender	αB-Crystallin	HSP27	HSP90
Human muscles					
*–*	(3×) Healthy CTRL	1953/72/78/M	Z-disc	Z-disc/Cytosol	Cytosol
*CAPN3*	LGMD2A^a^/Calpainopathy		I-band	I-band	I-band
	1) p.Thr184Argfs & Trp130 > Cys	1967/M			
	2) p.Thr184Argfs & p.Arg315Arg	1957/M			
	3) p.Thr184Argfs & p.Gly329Arg	1968/F			
*DMD*	Duchenne muscular dystrophy		I-band	I-band	I-band
	1) Del. exon 2–18	1998/M			
	2) Del. exon 3–11	2014/M			
*FLNC*	Filaminopathy (2×) p.Val930_Thr/933del	1960/M 1960/F	I-band	I-band	I-band
*TTN*	Titinopathy/HMERF^b^ (2×) p.Cys30071Arg	Unknown/Unknown	I-band	I-band	I-band
*MYOT*	Myotilinopathy		I-band	I-band	I-band
	1) p.Ser60Phe	1928/M			
	2) p.Lys36Glu	1940/F			
*VCP*	IBMPFD^c^ (Valosin-containing protein)		I-band	I-band	I-band
	1) p.Arg93Cys	1971/M			
	2) p.Arg155His	Unknown/M			
*DES*	Desminopathy p.Arg350Pro	1976/M	Cytosol	Cytosol	I-band/ Cytosol
*–*	(3×) Sporadic inclusion body myositis (sIBM)	1955/1998/2014/M	Z-disc	Z-disc/Cytosol	Cytosol
Mouse muscles					
*–*	Litter-matched WT CONTROLS	2016/M	Z-disc	Z-disc/Cytosol	Cytosol
*FLNC*	MFM filaminopathy p.W2711X	2016/M	I-band	I-band	I-band
*DMD*	mdx C57BL/10ScSn	2016/M	I-band	I-band	I-band

^a^LGMD2A, Limb girdle muscular dystrophy type 2A

^b^HMERF, Hereditary myopathy with early respiratory failure

^c^IBMPFD, Inclusion body myopathy with Paget disease and frontotemporal dementia

### Ethics, consent and permissions

Patients consented to participate in this study, which conforms to the principles outlined in the declaration of Helsinki and was approved by the ethics committee at Ruhr University Bochum (entries 3447–09 and 3483–09).

### Mouse models of hereditary myopathies

Skeletal muscle samples were obtained from two published mouse models of hereditary myopathies, the MFM-filaminopathy mouse (*FLNC*, p.W2711X; [12]) and the mdx mouse (C57BL/10ScSn), the latter of which was a kind gift from Dr. Jens Schmidt (Göttingen, Germany). Littermate wildtype (WT) mouse muscles served as controls. Four (mdx model) and six (FLNC model) animals per group, respectively, were studied.

### Passive tension measurements

Force measurements were done according to published protocols [51]) on isolated skinned muscle fibers from CTRL (2 subjects, 20 fibers), LGMD2A (2 subjects, 12 fibers) and MFM-filaminopathy (2 patients, 15 fibers) biopsies. Deep-frozen biopsy tissue was defrosted and skinned overnight in ice-cold low ionic-strength buffer (75 mM KCl, 10 mM Tris, 2 mM MgCl_2_, 2 mM EGTA, and 40 μg/ml protease inhibitor leupeptin, pH 7.2) supplemented with 0.5% Triton X-100. Under a binocular (Leica, Mannheim, Germany), single muscle fibers were dissected and suspended between two mini forceps attached to a piezomotor and a force transducer (Scientific Instruments, Heidelberg, Germany). Force measurements were carried out in relaxing buffer (8 mM ATP, 20 mM imidazole, 4 mM EGTA, 12 mM magnesium propionate, 97 mM potassium propionate, pH 7.2) at room temperature. Stretching of fibers was done stepwise from slack length in 6 quick steps. Following each step the fiber was held at a constant length for 60 s to allow for stress relaxation. After the last step-hold, the fiber was released back to slack length. Sarcomere length (SL) was measured by laser diffraction. Passive force-SL recordings were also performed in the presence of recombinant sHSP. Briefly, the 6-ramp step stretch protocol was first carried out twice in the absence of sHSP and then repeated twice in the presence of 100 μM αB-crystallin or HSP27 (10 μM sHSP showed no effect on PT). For data analysis we considered only the peak force levels at the end of each step, which represents a mixture of elastic and viscous forces. Force was related to the cross-sectional area inferred from the diameter of the specimens (at slack length), to obtain PT. Mean data points and SEM were calculated and fit with a simple polynomial. Some samples were chemically fixed (see below) immediately after mechanical measurements, usually at a stretched length, and studied for endogenous vs. exogenous sHSP localization by indirect immunofluorescence.

### Immunofluorescence microscopy

Muscle biopsy samples were fixed in 4% paraformaldehyde (PFA), 15% saturated picric acid in 100 mM phosphate buffered saline (PBS) overnight at 4 °C, dehydrated via ascending ethanol series and embedded in paraffin. Thin sections (5–7 μm) were cut with an RM 2235 Leica microtome (Mannheim, Germany). Sections were rehydrated, blocked in peroxidase blocking buffer, and a citrate-EGTA antigen recovery protocol was performed. Slides were rinsed with PBS and then blocked with 5% bovine serum albumin including 0.5% Triton X-100 for 60 min. Subsequently, sections were incubated with primary antibodies overnight at 4 °C, using one of the following antibodies against (all dilutions in PBS): HSP20 (ab125125, Abcam; 1:1000), HSP22 (ab151552, Abcam; 1:150), HSP27 (Clone 2B4, LSBio; 1:100), HSP27 (SMC 1615D12, StressMarq; 1:250), HSP27 (SR B800, MBL; 1:200), αB-crystallin (SMC 1653A10, StressMarq; 1:250), αB-crystallin (SR 223F, MBL; 1:400), HSP40 (Abcam, ab78437; 1:100), HSP70 (Novocastra/Leica; 1:20) HSP70 (HSPA2, Sigma HPA000798; 1:200), HSPA5 (78 kD glucose-regulated protein, Sigma, HPA038845; 1:500), Hsc70 (Heat shock cognate 71 kDa protein, Abcam, ab2788; 1:300), HSP90 (C45G5, Cell Signaling; 1:400), HSP90α (custom-made by PINEDA Berlin, 1:500), titin 9D10 PEVK (Hybridoma Bank, Iowa City, USA; 1:200), and titin custom-made against PEVK C-terminal segment (Eurogentec, polyclonal, affinity-purified; 1:500). Secondary antibodies were Cy3- or FITC-conjugated IgG (Rockland; 1:400), which were incubated overnight at 4 °C. For endogenous vs. exogenous HSP localization in mechanically stretched myofibers, we used a 6xHIS-conjugated IgG (Abcam ab9136; 1:400) antibody incubated overnight at 4 °C. Stained samples were embedded in Mowiol supplemented with N-propyl-gallate for bleaching protection and analyzed by confocal laser scanning microscopy (Nikon, Eclipse Ti), using a 63× oil Plan-Apochromat objective.

### Immunoelectron microscopy

4% PFA fixed biopsy samples were cut into longitudinal 50-μm-thick sections using a VT 1000S Leica vibratome (Mannheim, Germany) and rinsed twice in PBS. Then, the muscle samples were blocked in 20% normal goat serum (NGS) for 1 h and incubated with primary antibodies in PBS supplemented with 5% NGS overnight at 4 °C. In addition to some of the primary antibodies used for indirect immunofluorescence (see above section), we also used anti-titin antibodies T12 (1:100; [22]) and N2A (custom-made by Eurogentec, Belgium, 1:500; [37]). The sections were then triple washed with PBS and incubated with 1.4 nm gold-coupled secondary antibodies (Nanoprobes, Stony Brook, NY, USA) overnight at 4 °C. After extensive washing, all sections were postfixed in 1% glutaraldehyde for 10 min and after rinsing, sections were reacted with HQ Silver kit (Nanoprobes). After treatment with OsO_4_, samples were counterstained with uranyl acetate in 70% ETOH, dehydrated and embedded in Durcupan resin (Fluka, Switzerland). Resin blocks were made and ultrathin sections prepared with a Leica Ultracut S (Mannheim, Germany) and adsorbed onto glow-discharged Formvar-carbon-coated copper grids. Microscopy was performed on a Zeiss LEO 910 electron microscope and images were taken with a TRS sharpeye CCD Camera (Troendle, Moorenwies, Germany). For some images, we measured the nearest distance across the sarcomeric Z-disc between the mean epitope positions of αB-crystallin, HSP27, HSP90, titin T12, titin N2A, and titin PEVK antibodies using ImageJ, as described previously [38]. The distance between epitopes was plotted against SL, and data points for each antibody type were fit by two-order regression. At least 10 different cells and 30 sarcomeres per experimental condition were included in the analysis.

### SDS-PAGE and immunoblotting

Deep-frozen biopsy tissue was homogenized in modified Laemmli buffer, stored on ice for 10 min and subsequently boiled for 10 min at 97 °C. The protein concentration was determined by spectroscopy using Neuhoff standard protocols, SDS–PAGE was carried out using the Laemmli buffer system in slab gels containing 12.5% polyacrylamide. For immunoblot analysis the proteins were transferred onto nitro-cellulose membranes by semidry electroblotting. The blots were transiently stained with Ponceau S to monitor transfer efficiency, then washed with Tris-buffered saline, and incubated for 2 h with a primary antibody. Chromogenic blotting with alkaline phosphatase conjugated secondary antibodies with nitro-blue tetrazolium and 5-bromo-4-chloro-3′-indolyphosphate was used to visualize chaperone expression on Western blots, using the following antibodies: anti-HSP27 (SR B800, MBL; concentration, 1:100), anti-αB-crystallin (SR 223F, MBL; 1:200), anti-HSP90α (PINEDA Berlin; 1:1000), and anti-β-actin (AC-15 Sigma; 1:100). For measurements of titin:myosin heavy chain (MHC) ratio, homogenized skeletal muscle biopsy samples were analyzed by 2.5% SDS–PAGE, as described [46]. For titin phosphorylation analysis, 1.8% SDS–PAGE was performed as described [23]. Global titin phosphorylation was determined by anti-phosphoserine/−threonine antibodies (catalogue No. PP2551 (ECM Biosciences); Biotrend Chemicals, Cologne, Germany). To detect site-specific titin phosphorylation, we used custom-made affinity-purified phospho-serine specific antibodies against pS11878 and pS12022 in the PEVK domain of human titin (custom-made by Eurogentec, Belgium; 1:500). As secondary antibody, we used horseradish peroxidase-conjugated IgG (Acris Antibodies, Herford, Germany). For signal amplification we used the enhanced chemiluminescence Western blot detection kit (GE Healthcare). Staining was visualized using the LAS-4000 Image Reader (Fuji Science Imaging Systems) and densitometry was performed using the manufacturer’s MultiGauge analysis software or ImageQuantTL (GE Healthcare). The signal on the Coomassie-stained PVDF membrane served as a means to detect total protein load (in lieu of a reference protein in the titin size range), and immunoblot signals were normalized to the corresponding PVDF signals. Finally, mean signals obtained for diseased muscle tissues were indexed to signals measured in control muscles.

### Statistical analysis

Mean values of PT at a given sarcomere length were compared using two-tailed Student’s t-test. Normal distribution of data was a requirement, as was the passing of the equal variance test. Mean densitometric values obtained from stained gels/Western blots were indexed to the respective mean values of human CTRL muscles and compared using Bonferroni adjusted t-test following ANOVA.

## Results

### Passive tension is increased in LGMD2A and MFM-filaminopathy myofibers

**Fig. 1:**
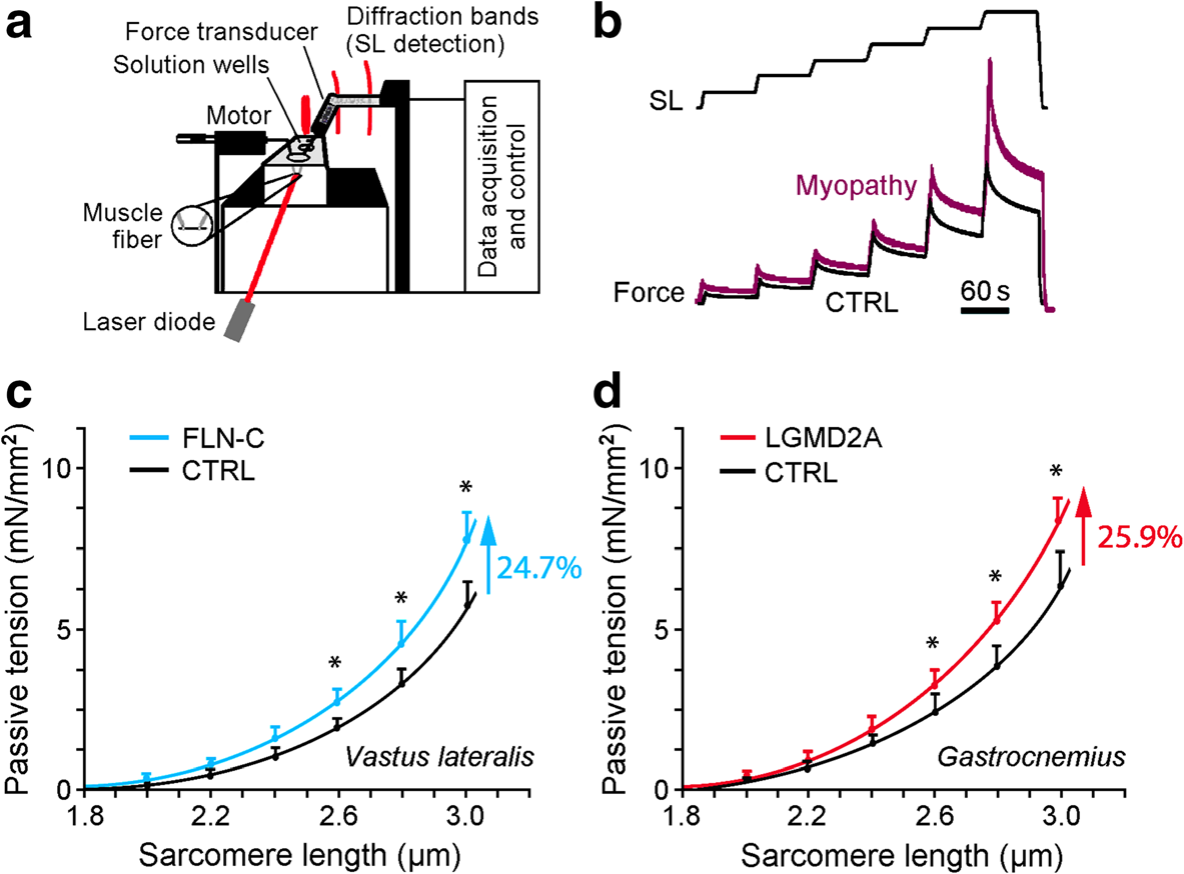
Passive sarcomere length-tension relationships of isolated normal and myopathic skinned myofibers. **(a)** Schematic of experimental setup. **(b)** Mechanical measurement protocol. Sarcomere length (SL) was increased stepwise from 1.8 to 3.0 μm. **(c)** Mean SL-dependent passive tension (PT) of normal (CTRL; *N* = 20 fibers) and MFM-filaminopathy (‘FLN-C’; *N* = 15 fibers) *vastus lateralis* muscle fibers, from two different individuals/patients per group. **(d)** Mean PT of normal (CTRL; *N* = 14 fibers) and LGMD2A (*N* = 11 fibers) *gastrocnemius* muscle fibers, from two different individuals/patients per group. Mean data points were fit with simple exponential functions. Symbols and error bars are means ± SEM; *p < 0.05 in Student’s t-test

We measured the SL-dependent PT in skinned normal and diseased myofibers from two different MFM-filaminopathy and LGMD2A patients, respectively (Fig. 1). A relaxed preparation was stretched from slack length to a series of desired SLs (maximum, 2.8–3.0 μm), and the peak PT in each step stretch was recorded (Fig. 1a, b). Fibers suspended in the mechanical setup showed no obvious differences in slack SL. For filaminopathy fibers we found up to 24.7% higher PT compared to CTRL fibers (Fig. 1c), for LGMD2A fibers up to 25.9% (Fig. 1d). For both groups, the difference was significant at SLs of 2.6 μm and above.

### Titin phosphorylation is reduced in dystrophic and MFM myofibers

**Fig. 2:**
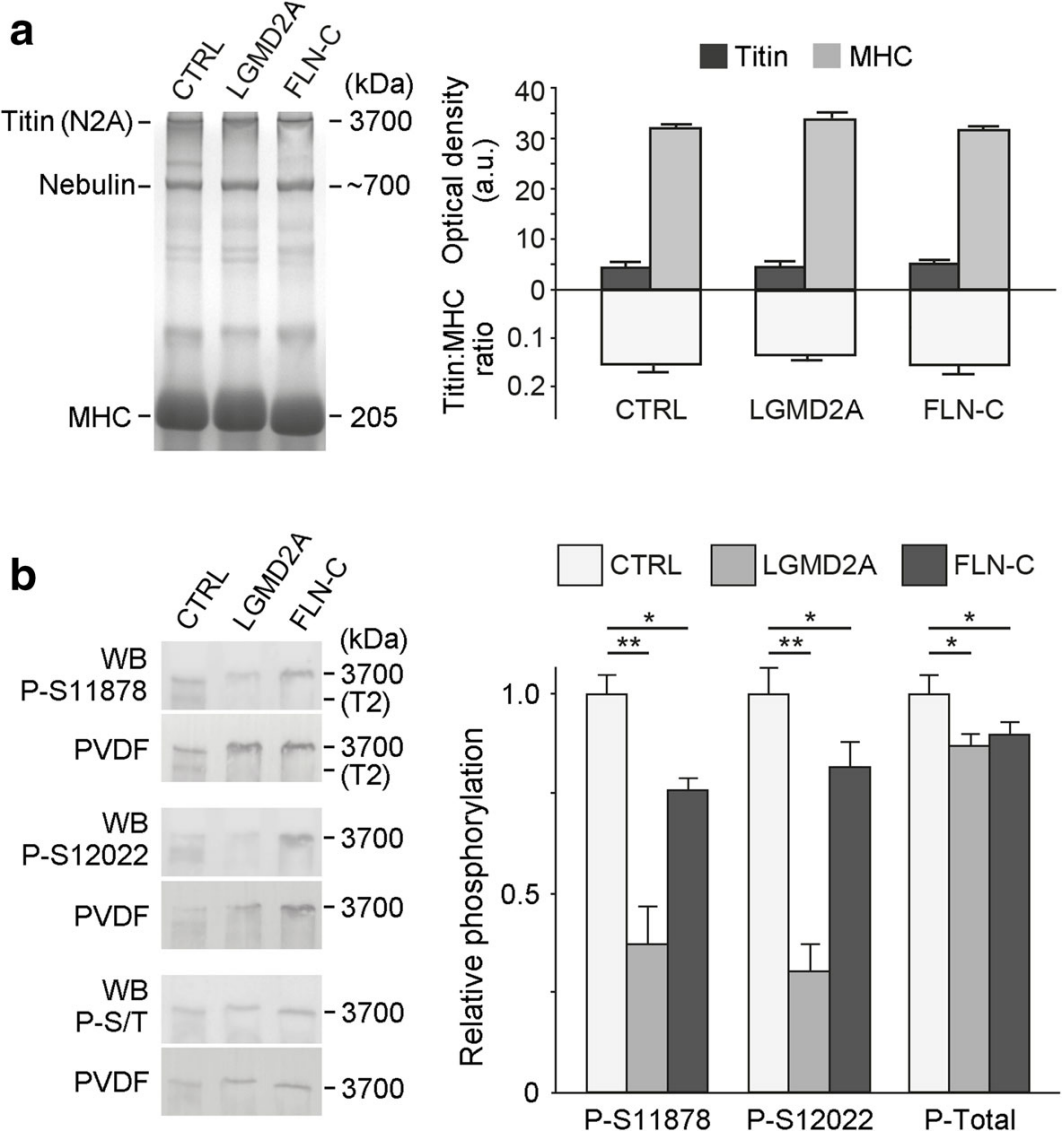
Titin isoform expression and phosphorylation in normal and diseased myofibers. **(a)** Representative SDS-polyacrylamide gels (left panel) to monitor titin molecular size (N2A isoform) and the titin:myosin heavy chain (MHC) expression ratio in normal (CTRL), LGMD2A, and MFM-filaminopathy (‘FLN-C’) muscles (*Vastus lateralis*). Right panel shows mean titin sizes and mean titin:MHC ratios. **(b)** Typical Western blots of titin bands on loose SDS-polyacrylamide gels (left panel) using phosphosite-specific antibodies to phospho-serines P-S11878 and P-S12022 in the PEVK domain of titin and to all phospho-serines/−threonines in titin (P-S/T). “PVDF” is the (transiently) coomassie-stained blotting membrane, the signal from which is shown in lieu of a loading control (not available in the titin size range). Right panel shows mean phosphorylation levels for CTRL, LGMD2A and MFM-filaminopathy muscles. Data are means ± SEM (*n* = 3/group); *p < 0.05, **p < 0.01, in Bonferroni-adjusted t-test

Because myofiber PT is largely determined by titin, we measured titin isoform expression and phosphorylation by titin gel electrophoresis and Western blot (Fig. 2). A comparison of FLN-C, LGMD2A, and human control muscles revealed the same molecular size of titin (~3.7 MDa, Fig. 2a). Moreover, the titin:MHC ratio also remained unaltered in myopathy (Fig. 2a), suggesting that the increased PT in diseased myofibers was not due to changes in titin expression levels. In contrast, both site-specific (PEVK) phosphorylation and global titin phosphorylation were significantly reduced in the two diseased muscle groups compared to CTRL muscle (Fig. 2 b). A lower-than-normal phosphorylation state of the PEVK titin region at the two sites studied here is known to reduce titin-based PT [25]. Thus, the altered titin phosphorylation state found in the diseased muscles is unlikely to account for the PT increase of these myofibers.

### Heat shock proteins translocate to the titin springs in different myopathies

Ultrastructural studies of human *Vastus lateralis* myocytes by electron microscopy revealed worsening of myofibrillar integrity and massive mitochondrial swelling in patient muscles, compared to normal human control muscles (Additional file 1: Figure S1a). At higher magnification, a peculiar increase in electron density was observed in the diseased samples at the sarcomeric I-bands on either side of the Z-discs, which was not seen in healthy controls. This finding implicated massive binding of proteins to the I-band, probably to the titin springs, as part of the pathology of these skeletal myopathies. We assumed that these proteins could be chaperones, such as sHSPs (HSP27, αB-crystallin) and HSP90, which are known to associate with I-band titin under stress [15, 31]. The expression levels of the chaperones were measured in muscle tissue from controls and five hereditary myopathies with mutations in *CAPN3* (LGMD2A), *FLNC* (MFM-filaminopathy), *DMD* (Duchenne muscular dystrophy), *MYOT* (MFM-myotilinopathy) or *VCP* (IBMPFD). We found that HSP27 and αB-crystallin were significantly increased, by a factor of 4–7, in all myopathy types, whereas HSP90 remained unaltered within experimental error, at best showing a trend for elevated expression in myopathy (Additional file 1: Figure S1b).

Next, we performed immunocytochemical analyses using antibodies against various HSPs, in order to identify the chaperones that were translocated to the I-band region in diseased myocytes. On immunoelectron micrographs, we observed HSP20, HSPB8, HSP70 and HSC70 to be mainly at the Z-discs and in part in the cytosol (Additional file 1: Figure S2). HSP40 was found mainly in the intermyofibrillar space (presumably at the sarcoplasmic reticulum), HSPA5 consistently perinuclear, BAG3 at Z-discs in between myofibrils, and Stub1 at the Z-disc and in the I-band. Importantly, all these chaperones did not differ in their intracellular localization between CTRL, LGMD2A (Additional file 1: Figure S2) and MFM-filaminopathy (not shown). Hence, these chaperones were considered unlikely to cause the rise in titin-based PT in skeletal myopathies and not studied further.

**Fig. 3:**
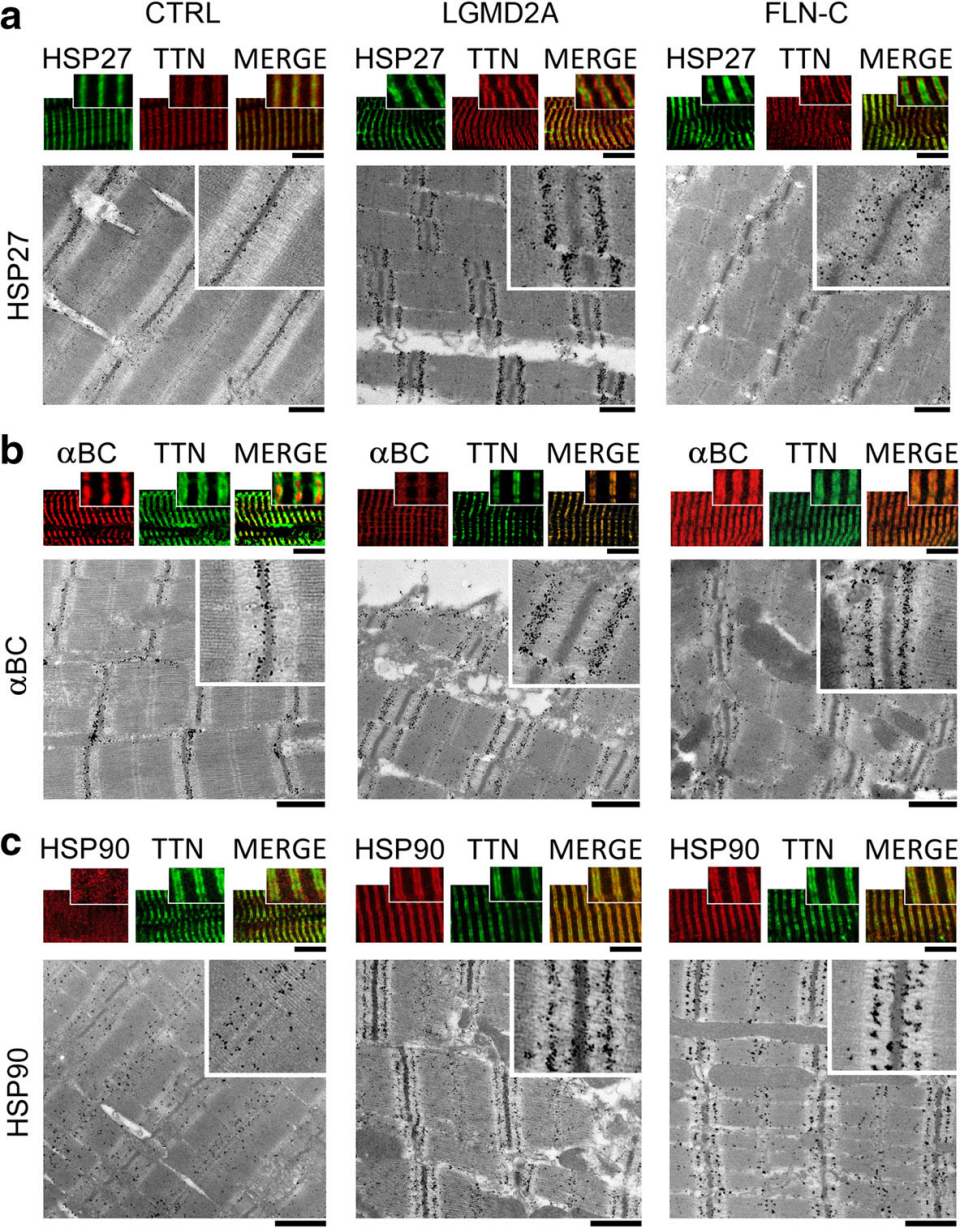
Correlative immunofluorescence and immunoelectron microscopy to localize HSP27, αB-crystallin and HSP90 in normal and diseased myofibers.** (a)** HSP27 localization on myofibrils. Top panels: representative immunofluorescence images of CTRL, LGMD2A and MFM-filaminopathy (‘FLN-C’) muscle cells labeled with antibodies to HSP27 (secondary antibody: FITC-conjugated IgG). Samples were counterstained against the PEVK titin (TTN) epitope (secondary antibody: Cy3-conjugated IgG); the merged image is on the right in each group. Bottom panels show nanogold-labeled immunoelectron micrographs. Insets, higher-power images of sarcomeric regions immunostained with anti-HSP27/anti-PEVK. **(b)** Localization of αB-crystallin (αBC). Top panels: immunofluorescence images of myofibers labeled with anti-αB-crystallin (secondary antibody: Cy3-conjugated IgG) and counterstained for PEVK titin (secondary antibody: FITC-conjugated IgG). Bottom panels show immunoelectron micrographs, insets magnifications. **(c)** Localization of HSP90. Top panels: immunofluorescence images of myofibers labeled with anti-HSP90 (secondary antibody: Cy3-conjugated IgG) and counterstaining for PEVK titin (secondary antibody: FITC-conjugated IgG). Bottom panels show immunoelectron micrographs, insets magnifications. Bars, 5 μm (confocal images) and 1 μm (EM). For a quantitation of nanogold particle distribution on these and similar immunoelectron micrographs, see Additional file 1: Figure S3

In contrast, the sHSPs HSP27 and αB-crystallin, as well as ATP-dependent HSP90, showed strongly altered localization in diseased muscle cells (Fig. 3). Typical immunofluorescence micrographs of CTRL myocytes revealed endogenous HSP27 preferentially at the Z-discs, localizing in-between the PEVK-titin epitope (Fig. 3 a). This intracellular localization was confirmed by immuno-EM. However, in LGMD2A muscle cells, the confocal analysis suggested specific HSP27 immunoreactivity in the sarcomeric I-bands close to the PEVK epitope. Immunoelectron microscopy using anti-HSP27 antibodies showed massive labelling of the I-band on either side of the Z-disc, whereas the Z-disc itself was barely stained (Fig. 3 a). Similarly strong HSP27 signals at the elastic I-band segment were obtained in MFM-filaminopathy (‘FLN-C’) biopsy samples. Quantitation of the gold particle distribution indicative of HSP27 protein (*n* = 30 sarcomeres) demonstrated that 60–70% of all nanoparticles were localized to the I-band away from the Z-disc in either myopathy type (Additional file 1: Figure S3a).

Staining for endogenous αB-crystallin and counterstaining for PEVK titin in CTRL muscles revealed preferential Z-disc localization of this sHSP and additional reactivity in the myofibrillar vicinity near the Z-discs (Fig. 3b). In both LGMD2A and MFM-filaminopathy patient muscles, αB-crystallin was at the elastic I-band region, localizing to a segment in between the Z-disc and (relatively close to) the PEVK titin epitope. Quantitation of the gold particle distribution on immunoelectron micrographs (*n* = 30 sarcomeres) verified that the vast majority of αB-crystallin (60–70% of gold particles) was in the elastic I-band in myopathy fibers, presumably bound to the titin springs (Additional file 1: Figure S3 b).

Staining for endogenous HSP90 in CTRL myocytes demonstrated a broad cytosolic distribution of the chaperone, with some clustering at the sarcomeric A-bands, where it likely binds to myosin (Fig. 3c). In both LGMD2A and MFM-filaminopathy muscles, HSP90 was abundantly found at the sarcomeric I-bands very near the PEVK titin epitope. Analysis of the gold particle distribution indicative of HSP90 protein (n = 30 sarcomeres) confirmed the preferential I-band binding (~50% of gold particles) in myopathy and also showed additional minor associations with the A-band (25%) and (only in filaminopathy muscles) the Z-disc (22%) (Additional file 1: Figure S3 c).

### Heat shock proteins associate with elastic titin at different I-band positions

**Fig. 4:**
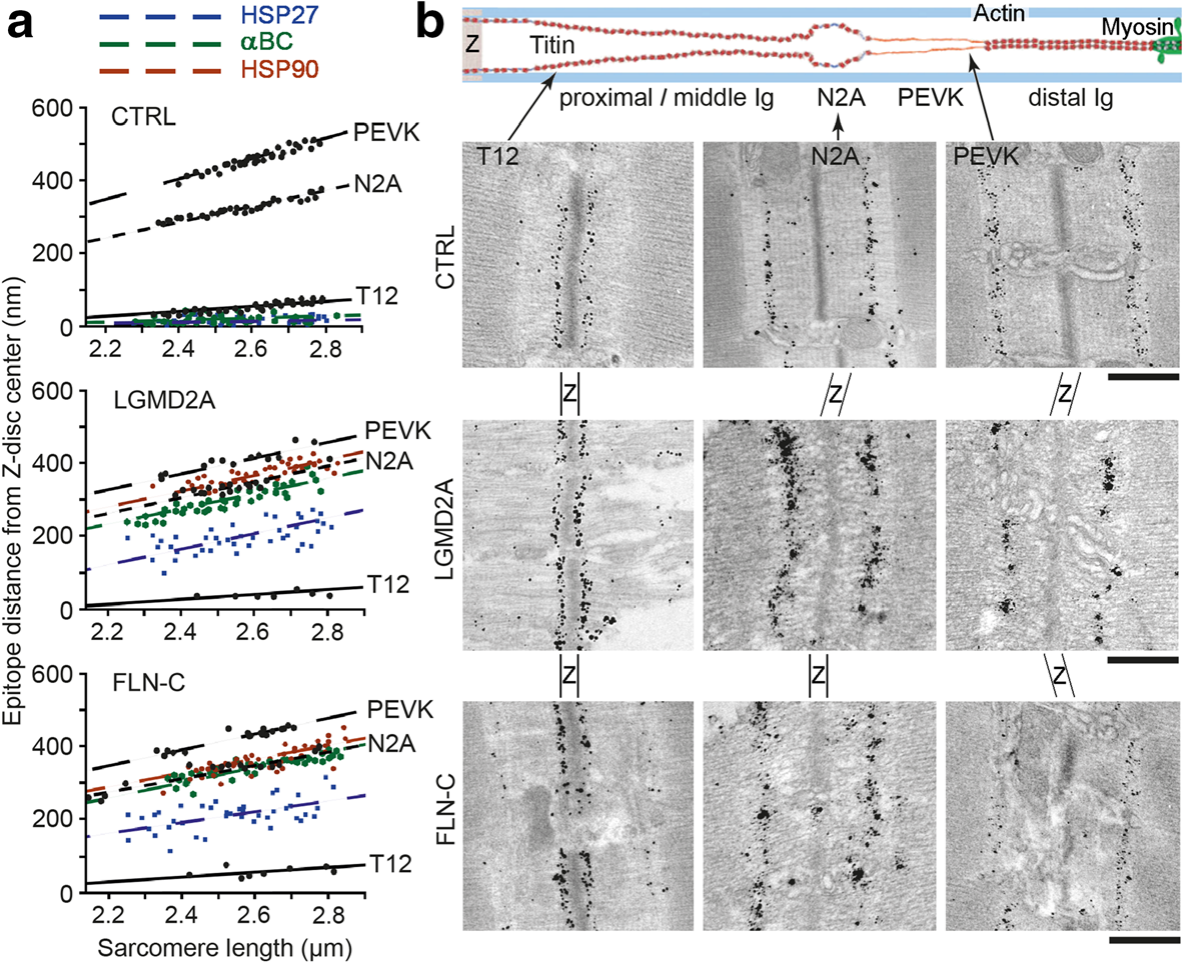
Sarcomeric binding sites of chaperones relative to I-band titin epitopes**. (a)** Nearest epitope-to-mid-Z-disc distance of gold particles indicating the position of sarcomere-bound HSP27, αB-crystallin (αBC) and HSP90, in comparison to that of the T12, N2A and PEVK titin epitopes, on immunoelectron micrographs of CTRL, LGMD2A, and MFM-filaminopathy (‘FLN-C’) myofibers at different sarcomere lengths. *N* = 30 sarcomeres analyzed per antibody and group. Lines are linear regression. **(b)** Schematic on top shows the domain architecture of I-band titin (N2A isoform) and the positions of anti-titin antibodies used (T12, N2A, PEVK). Immunoelectron micrographs below were taken from CTRL and diseased human skeletal muscle biopsy samples labeled with anti-titin T12, N2A and PEVK antibodies, respectively. Z, Z-disc region

To elucidate the precise binding sites of the three chaperones along I-band titin, we compared the SL-dependent epitope positions of anti-HSP antibodies in the sarcomere with those of different I-band titin antibodies (Fig. 4). To this end, immunoelectron microscopy was performed on CTRL, LGMD2A, and MFM-filaminopathy (‘FLN-C’) muscles using the T12 (start of proximal Ig-domain segment), N2A, and (C-terminus of) PEVK anti-titin antibodies (Fig. 4b). In CTRL cells, HSP27 and αB-crystallin were localized in-between the Z-disc center and the T12 epitope (Fig. 4a, top), confirming their Z-disc association. Because of the diffuse cytosolic localization of HSP90 in CTRL cells, a distinct epitope position was not defined for this chaperone. In LGMD2A and MFM-filaminopathy myocytes, HSP27 was localized rather broadly in-between the T12 and N2A epitopes, apparently bound to the proximal/middle titin Ig-domain segment, whereas αB-crystallin was at a sarcomeric I-band location farther away from the Z-disc, near the N2A region (Fig. 4a). HSP90 was also localized in proximity to the N2A element, slightly closer to but never reaching the PEVK domain. Importantly, all chaperone binding sites translocated away from the Z-disc with increasing SL, indicating that the HSPs bind to the elastic titin springs (and not to the thin filaments).

### HSP binding to elastic titin is common in hereditary myopathies, but not acquired sIBM

**Fig. 5:**
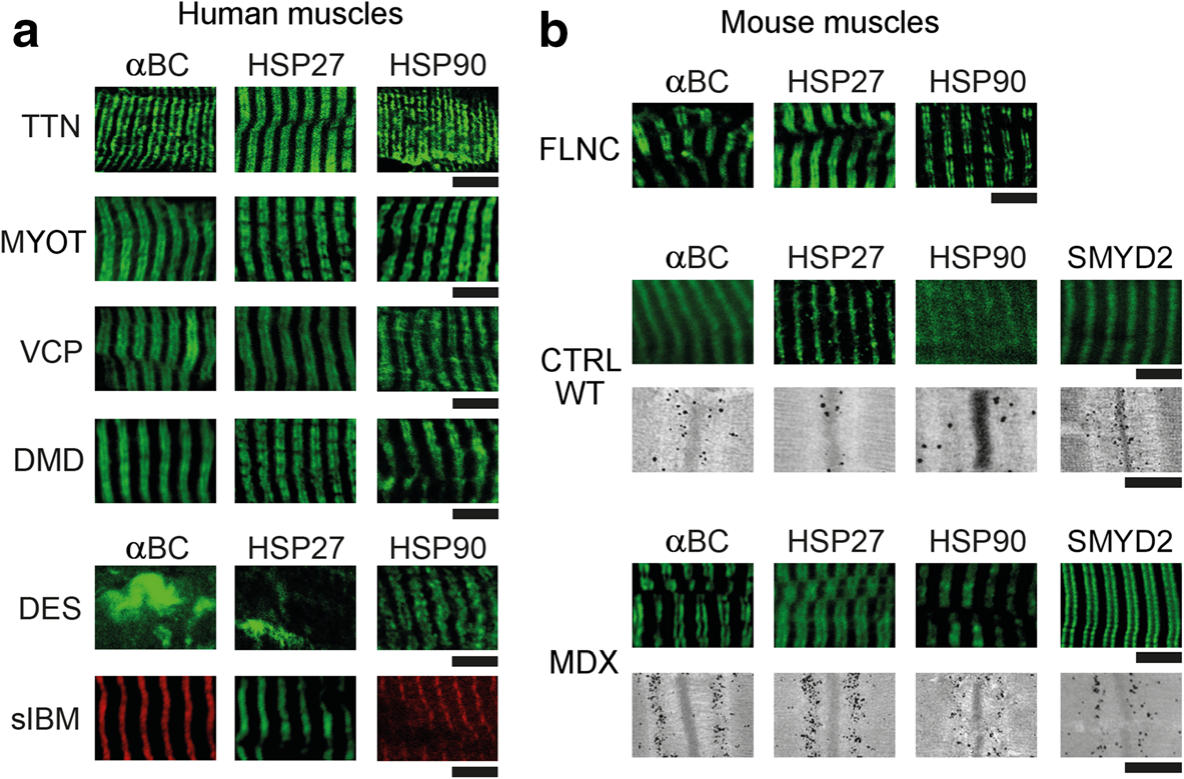
Localization of sHSPs and HSP90 in myofibers of additional human myopathies and mouse models.** (a)** Sarcomeric I-band association of αB-crystallin (αBC), HSP27 and HSP90 in human HMERF-titinopathy (TTN), MFM-myotilinopathy (MYOT), IBMPFD due to mutations in VCP, and DMD, by indirect immunofluorescence (secondary antibodies: FITC-conjugated IgG or Cy3-conjugated IgG). In human MFM-desminopathy (DES) only HSP90 was I-band-associated, whereas the two sHSPs were found mainly in cytosolic aggregates. In sporadic inclusion body myositis (sIBM), the chaperones were detected in the cytosol and at the Z-disc (as in CTRL muscles). Note that in healthy CTRL myofibers (not shown here), the chaperones were always at the Z-disc and in the cytosol and usually did not show the doublet banding pattern typical for most myopathies. For additional immunostainings on these human samples, and counterstaining with anti-titin, see Additional file 1: Figure S4. **(b)** Sarcomeric I-band association of αB-crystallin, HSP27 and HSP90 in mouse models of MFM-filaminopathy (FLNC) and Duchenne muscular dystrophy (MDX), as well as normal CTRL muscles, by indirect immunofluorescence (secondary antibodies: FITC-conjugated IgG) and nanogold-labeling immuno-EM. CTRL and MDX myofibers were also immunostained against methyltransferase Smyd2 (co-chaperone of HSP90). For additional immunostainings on these mouse samples, and counterstaining with anti-titin, see Additional file 1: Figure S5. Bars, 5 μm (confocal images) and 1 μm (EM)

We wanted to know whether the observed translocation of HSPs indicates a more general pathological response in skeletal myopathies. Hence we immunostained additional muscle biopsies from patients with other hereditary dystrophic or MFM muscle disorders, including HMERF-titinopathy, DMD, Myotilinopathy, IBMPFD due to mutation in VCP, and Desminopathy (Fig. 5a and Additional file 1: Figure S4). For comparison, we added acquired sporadic inclusion body myositis (sIBM). We found that HSP27, αB-crystallin and HSP90 were translocated to the I-band titin springs in all hereditary myopathies, with the exception of Desminopathy. In the latter, the two sHSPs were detected mainly in cytosolic aggregates, whereas HSP90 was again found in the elastic I-band region (Fig. 5a and Additional file 1: Figure S4 e). In the three different sIBM biopsies studied, none of the chaperones showed the I-band localization typical of hereditary myopathies. In these sIBM muscles, the staining pattern was similar to that in CTRL muscles, with positive immunoreactivities in the cytosol and/or Z-disc (Fig. 5a and Additional file 1: Figure S4 f). Taken together, translocation of HSPs to the I-bands was observed in 13 patients and 6 different types of hereditary myopathies, but not in healthy control and acquired idiopathic inflammatory myopathy muscles (Table 1).

### HSP binding to elastic titin is also seen in animal models of inherited myopathies

In addition, we carried out HSP-localization experiments in two established animal models of hereditary myopathies, the dystrophic mdx (mutant *DMD*) and the FLNC (p.W2711X) MFM-filaminopathy mice, as well as in the respective littermate CTRL WT muscles. As in the human muscles, HSP27, αB-crystallin and HSP90 were translocated to the sarcomeric I-bands in both myopathy models, whereas the chaperones appeared at the Z-disc and in the cytosol in WT (Fig. 5b and Additional file 1: Figure S5 a-c). In an attempt to identify possible triggers for the binding of HSP90 (which has a myriad of clients) to I-band titin, we also immunostained CTRL WT and mdx mouse muscles for Smyd2, an enzyme known to methylate HSP90 as a prerequisite for the association of the Smyd2-methyl-HSP90 complex with the N2A titin region [15]. We detected Smyd2 mainly at the Z-discs and in the cytosol of WT myocytes, but at the titin springs near the PEVK epitope in mdx muscle cells, resembling the staining pattern of HSP90 (Fig. 5b and Additional file 1: Figure S5 d).

### Incubation of skinned human myofibers with sHSPs can increase their passive tension

**Fig. 6:**
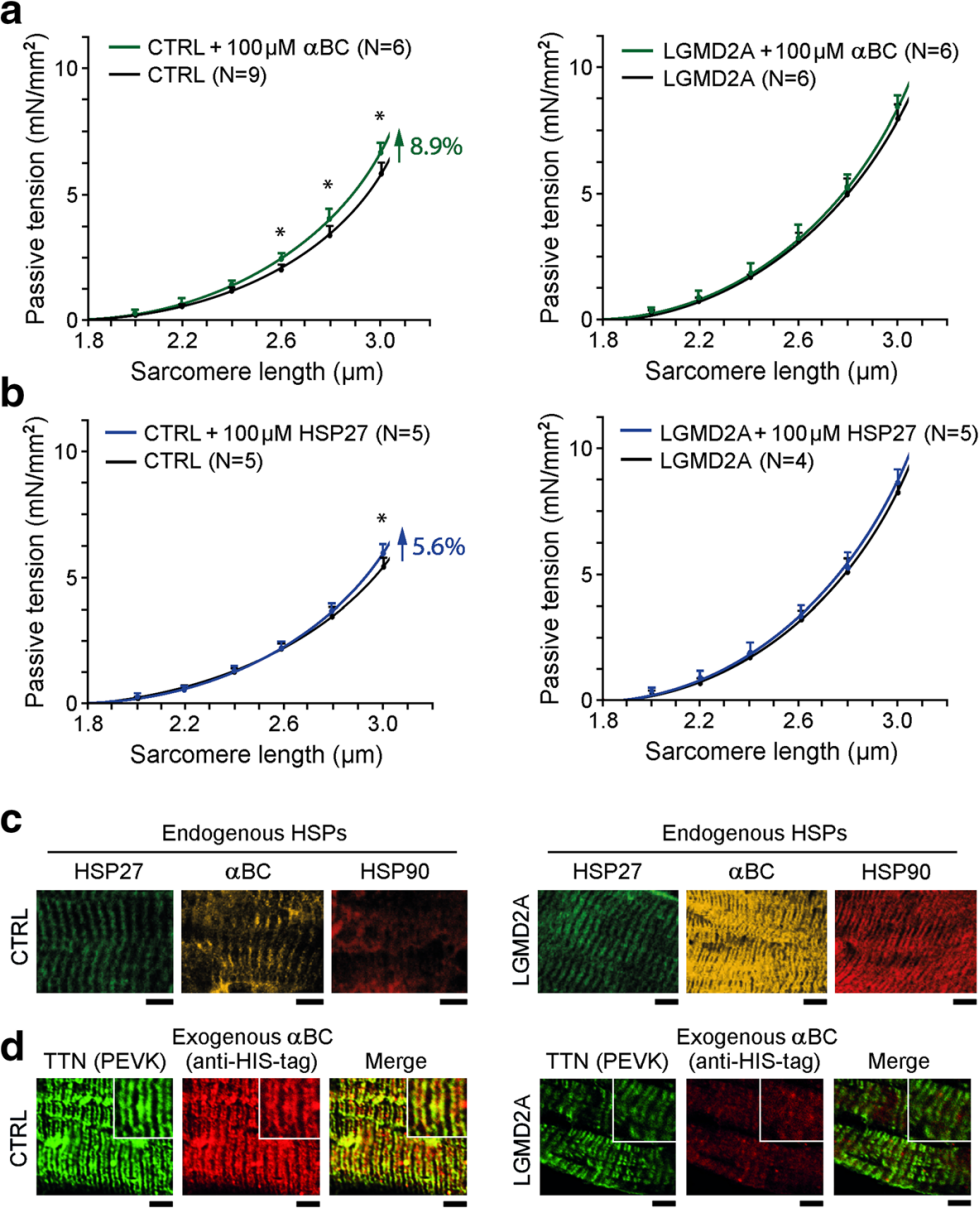
Passive tension of skinned normal and myopathy myofibers in the presence of recombinant sHSPs. **(a)** and **(b)** Passive sarcomere length-tension relationships of *Vastus lateralis* muscle fibers from CTRL (left panels) and LGMD2A patients (right panels), before and during incubation with (a) αB-crystallin (αBC) or (b) HSP27 recombinant protein (100 μM). Data points are means ± SEM. The number of fibers measured for each condition (N) is indicated; fibers were obtained from 2 subjects/group. Curves are polynomial fits to the means. *p < 0.05 in Student’s t-test. **(c)** Localization of endogenous HSP27, αB-crystallin and HSP90 in skinned myofibers after force measurements, monitored by indirect immunofluorescence microscopy. Left panels, CTRL myofibers; right panels, LGMD2A myofibers. **(d)** Localization of exogenous (6xHIS-tagged) recombinant αB-crystallin, in relation to the PEVK titin epitope (TTN), measured using anti-6xHIS-tag Cy3-conjugated antibodies. Left panels, CTRL myofibers; right panels, LGMD2A myofibers. Insets: Higher-power images of regions-of-interest. Muscle samples were fixed in the stretched state after mechanical measurements and incubated with the respective antibodies. All bars, 5 μm

Finally, we tested whether the binding of HSPs to elastic titin could account for the increased PT seen in diseased muscle fibers (Fig. 1). Accordingly, we measured the SL-PT curve of skinned single myofibers from CTRL and LGMD2A patients in the mechanical setup, before and after incubation with HSP27 or αB-crystallin recombinant protein (Fig. 6a, b). Following these mechanical measurements, some fibers were prepared for indirect immunofluorescence, to measure both endogenous HSPs (Fig. 6c) and — using anti-HIS-tag antibody to recombinant, HIS-tagged αB-crystallin — exogenous αB-crystallin, the latter also in comparison to the PEVK epitope (Fig. 6d). We found that in this subset of fibers used for mechanical measurements, CTRL and LGMD2A samples showed similar amounts of endogenous HSP27 localized to the Z-disc/I-band region (Fig. 6c). In contrast, endogenous αB-crystallin was much more abundant in LGMD2A than in CTRL, localizing massively to the I-band region. Because of these differences, exogenous (recombinant) αB-crystallin could abundantly bind to the I-band region in CTRL, whereas it was barely detectable at the sarcomeres in LGMD2A (Fig. 6d). Consistent with these findings, we observed only very little effect of incubation with recombinant HSP27 on PT, in both CTRL and LGMD2A myofibers (Fig. 6b). However, incubation with αB-crystallin increased the PT of CTRL fibers on average by nearly 10%, whereas the PT of LGMD2A fibers remained unaltered by this treatment (Fig. 6a). Collectively, these results suggest that sHSP-binding to elastic titin regions can increase the PT of the myofibers and explain, in part, the pathologically elevated PT of muscle cells found in LGMD2A and MFM-filaminopathy patients.

## Discussion

Increased muscle stiffness is frequently seen in patients with acquired or inherited myopathies, next to muscle weakness and atrophy as the main symptoms in these disorders. Pathological increases in passive muscle stiffness were observed in DMD patients [14, 32, 33] and greatly elevated myofiber PT was reported for patients with spasticity caused by impairment of the central nervous system [20, 49] or patients with facio-scapulohumeral muscular dystrophy [35]. However, the mechanisms behind these alterations in passive mechanical properties have remained largely unknown. Here, we measured the PT of isolated myofibers from two groups of hereditary myopathies, LGMD2A and MFM-filaminopathy patients, and detected approximately 25% higher levels in either group compared to healthy human myofibers. We excluded titin-isoform transitions and titin phosphorylation changes as causes of this increase. Instead, we found that the PT rise in myopathic muscles is due, at least in part, to intracellular translocation of chaperones to the sarcomeric titin springs, which were devoid of chaperones in healthy muscles. The HSPs that were translocated to I-band titin in myopathy included the two sHSPs HSP27 and αB-crystallin and the ATP-dependent chaperone HSP90. Importantly, we demonstrated that binding of these chaperones to elastic titin is common to hereditary skeletal muscle disorders, but not in acquired human sIBM. We found that the I-band titin-binding pattern of chaperones also appears in mouse models of dystrophic and MFM myopathies, but not in normal WT mouse muscles.

HSP27 and αB-crystallin were shown earlier to translocate to the sarcomeric Z-disc/I-band region of skeletal myofibers under stress conditions. The diverse stressors included intense exercise [50], myofibril stretching [31] or disease conditions, such as neurogenic atrophy and central core disease [18]. The cause of this translocation is incompletely understood. Potential triggers could be intracellular oxidative stress and acidosis [3, 34], possibly resulting from massive mitochondrial alterations, such as those observed by us in all myopathy samples. Acidic conditions directly affect the sHSPs by promoting the formation and accumulation of large oligomers, thereby increasing chaperone activity [11, 17]. While reduced pH boosts the aggregation of many sHSP substrates, it also increases the sHSP-mediated protection from aggregation [3, 6]. Interestingly, acidosis raises the passive stiffness of skeletal muscles [44]. Although intracellular pH and oxidative stress were not measured in our biopsy samples, it is reasonable to speculate that exercise increases these parameters more in myopathic than in healthy muscles, which may then cause higher chaperone activity (possibly towards elastic titin) in the diseased cells.

A likely trigger for the translocation is the increased expression of sHSPs, which is typical for skeletal myocytes exposed to different stresses [30, 50]. High levels of sHSPs are beneficial, as they protect cells from oxidative stress, acidosis, energy depletion, and other unfavorable conditions [45]. In the hereditary dystrophic and MFM muscles studied by us, the expression levels of HSP27 and αB-crystallin were much higher than in normal control muscles. Consequently, the sarcomeres could represent a “sink” for excessive amounts of sHSPs expressed in the diseased myocytes. A proportion of this surplus of chaperone protein may be trapped by “sticky” hydrophobic regions of the sarcomeric I-bands.

We recently showed that sarcomere stretching promotes the unfolding of titin Ig domains in the I-band [52], which results in the exposure of previously concealed hydrophobic titin regions, to which the sHSPs preferentially bind [31]. The phosphorylation state of the sHSPs, known to be relevant for their interaction with some substrates, does not seem to alter the interaction with titin domains [19, 31]. In the LGMD2A and MFM-filaminopathy samples studied in the present work, we detected HSP27 spread out along the proximal/middle tandem-Ig segment of I-band titin. This segment contains many relatively weak domains that unfold under physiological stretch forces [52]. AlphaB-crystallin was found to be restricted to a narrower region near/at the N2A element of titin, which also comprises Ig domains. In contrast, the sHSP-binding spared titin’s PEVK domain, a permanently unfolded (disordered) region, and the distal tandem-Ig region, which contains more stable Ig domains that rarely unfold under physiological stretch forces [52]. Assuming that sHSP-binding to the sarcomeric I-bands may be an indirect measure of the unfolded state of the titin Ig domains, our findings implicate increased unfolding of proximal/middle Ig domains in hereditary myopathy patients, possibly due to higher I-band strain than in normal myofibers. In summary, the increased association of sHSPs with the sarcomeric I-bands in myofibers of hereditary myopathy patients likely reflects increased interaction with unfolded titin Ig domains.

Conceptually, unfolding of the proximal/middle Ig domains of I-band titin raises their risk for irreversible aggregation, whereas sHSP-binding lowers this risk and protects the sarcomere [31]. Small HSPs are known to capture up to an equal weight of (partially) denatured protein before it aggregates [5]. Thus, sHSPs keep the substrate accessible to other members of the protein quality-control network, notably ATP-dependent chaperones, which are required for subsequent substrate refolding [42]. If refolding to the native state is not possible, the substrate is likely to be degraded. Hence, the binding of sHSPs to titin Ig domains could maintain the domains in a state that allows their efficient refolding. However, the binding could also be indicative of increased titin protein degradation and turnover in myopathic fibers. Either way, the sHSPs will have an important role in avoiding titin loss-of-function and preserving sarcomeric and muscle functions.

Interestingly, among the proteins overrepresented in protein aggregates of three MFM types (myotilinopathy, desminopathy and filaminopathy), there were many sarcomeric and other cytoskeletal proteins, especially Z-disc proteins, as well as various heat shock proteins (including HSP27 and αB-crystallin), but not titin [28, 40, 41]. In light of our results, it appears that misfolded/aberrant and potentially toxic titin is not “disposed” in aggregates, like many other cytoskeletal proteins in MFM. Instead, the sHSPs may help maintain titin in the sarcomere in a (partially) functional state, in order to preserve its role as the backbone of the sarcomere in the diseased myocyte. A deviation from this pattern of titin protection by sHSPs was observed only in the single desminopathy patient studied by us. In this biopsy sample, both HSP27 and αB-crystallin were mainly found in aggregates, the defining pathological features of this MFM. Currently we do not know why this patient muscle lacked the I-band binding pattern of sHSPs characteristic of the other myopathy types. Additional desminopathy patient samples should be studied to address this issue.

The presumed protective effect of the sHSPs on titin in most dystrophic and MFM disorders comes at the price of modestly increased passive muscle stiffness. This was suggested by the higher myofiber PT following binding of exogenous sHSPs to elastic titin in controls, but not LGMD2A fibers (which had higher PT than controls before the incubation with sHSPs). Moreover, the sHSPs can interact with and stabilize the folded Ig domains of the titin spring, which would further increase titin-based PT [9]. Because titin-dependent PT modulates the active contractile properties of skeletal myofibers [26, 36, 52], the increased PT observed in human myopathy presumably affects, to some degree, the developed tension of patient muscles. We conclude that there is a trade-off between beneficial (protection of unfolded protein) and detrimental effects (mechanical impairment) of sHSP-binding to I-band titin on sarcomere function, with consequences for overall muscle performance in myopathy.

Apart from the sHSPs, we studied a set of other chaperones for their intracellular localization in myopathic versus control muscles. However, the only chaperone that also showed a differential binding pattern was HSP90. This ATP-dependent HSP was mainly in the cytosol in controls and translocated to I-band titin in all hereditary dystrophic and MFM human samples, as well as in the DMD and MFM-filaminopathy mouse models, but not in acquired sIBM. Up to half of the cytoplasmic pool of HSP90 was associated with elastic titin (at the position of the N2A element) in human LGMD2A and MFM-filaminopathy myofibers. This binding pattern might indicate increased proteasomal degradation of I-band titin fragments. Importantly, HSP90 did not show significantly altered expression levels in the diseased muscles. Thus, a negative effect of the massive translocation of HSP90 to the titin springs could be the lack of this chaperone in myocyte compartments where it is usually required to perform its many chaperoning tasks. These findings support a concept [8] whereby the induction of HSP90 in myopathic muscles could be useful to ameliorate some pathological features in the patients.

A mechanistic link between HSP90 and sarcomeric proteins is evident from previous work. HSP90 is known as an essential modulator of myofibril organization and thick filament assembly [4, 13, 24]. Although much of the evidence is related to its association with myosin, HSP90 also binds to the N2A element of titin if methylated by a co-chaperone, the methyltransferase Smyd2 [15]. The Smyd2–methyl-HSP90 complex stabilizes the N2A element and helps maintain the sarcomeric Z-disc/I-band structure, which benefits muscle contraction [15, 58]. Because methylation of HSP90 by Smyd2 could be a trigger for the translocation of HSP90 to elastic titin in hereditary myopathies, we included Smyd2 in our immunoelectron/immunofluorescence microscopical analysis of WT and mdx mouse muscles. We found that Smyd2 translocated from the cytosol and Z-disc region in CTRL myocytes to the site of the titin N2A element in mdx, just as did HSP90. Thus, Smyd2 might guide HSP90 to the titin filaments in the sarcomeres, where the chaperone would then exert protective functions on the N2A element and possibly, other I-band proteins. In conclusion, while the functional consequences of the translocation of HSP90 to elastic titin in hereditary myopathies remain speculative, this chaperone binds to sub-sarcomeric sites other than those that associate with sHSPs, which implicates different protective functions.

## Conclusion

In summary, this study used high-resolution correlative microscopy to demonstrate the sub-cellular re-distribution of members of the sHSP family and HSP90 in diseased versus healthy muscles. We provided evidence for a putative chaperone-mediated protective mechanism involved in the maintenance of the sarcomeric I-band in hereditary, but not acquired myopathies. In a panel of human muscles with dystrophic and MFM disorders due to mutations in specific genes, we found HSP27, αB-crystallin, and HSP90 to bind in a non-redundant manner to elastic titin regions, presumably to prevent unfolded titin Ig domains from aggregating and to exert additional protective functions. The interaction of sHSPs with the titin springs caused increased myofiber PT, which may be detrimental to dystrophic and MFM patients. Thus, the HSP-binding to titin is a novel phenomenon common to hereditary myopathies, which could be in place to preserve the structural integrity and function of the contractile apparatus in skeletal muscle cells.

## Additional file

Additional file 1: This file contains Figures S1 to S5 and the corresponding figure legends. (PPTX 17980 kb)
