# Supplementary material for: Translocation of molecular chaperones to the titin springs is common in skeletal myopathy patients and affects sarcomere function
Source: Acta Neuropathol Commun. 2017 Sep 15;5:72. doi: 10.1186/s40478-017-0474-0 (PMC5603016; doi:10.1186/s40478-017-0474-0)
Supplement: Additional file 1: — This file contains Figures S1 to S5 and the corresponding figure legends. (PPTX 17980 kb) [file 40478_2017_474_MOESM1_ESM.pptx]

## Slide 1
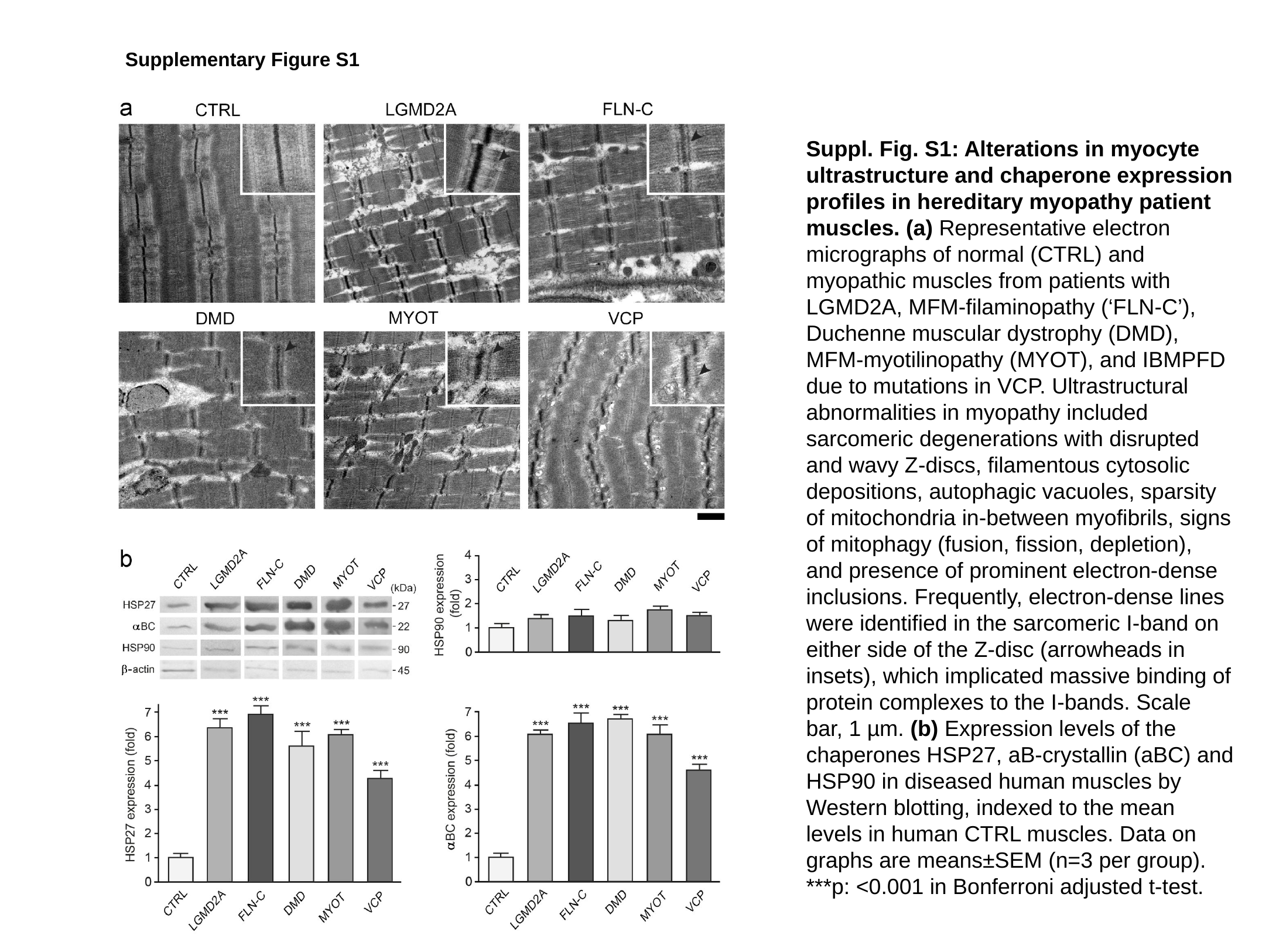

Supplementary Figure S1
Suppl. Fig. S1: Alterations in myocyte ultrastructure and chaperone expression profiles in hereditary myopathy patient muscles. (a) Representative electron micrographs of normal (CTRL) and myopathic muscles from patients with LGMD2A, MFM-filaminopathy (‘FLN-C’), Duchenne muscular dystrophy (DMD), MFM-myotilinopathy (MYOT), and IBMPFD due to mutations in VCP. Ultrastructural abnormalities in myopathy included sarcomeric degenerations with disrupted and wavy Z-discs, filamentous cytosolic depositions, autophagic vacuoles, sparsity of mitochondria in-between myofibrils, signs of mitophagy (fusion, fission, depletion), and presence of prominent electron-dense inclusions. Frequently, electron-dense lines were identified in the sarcomeric I-band on either side of the Z-disc (arrowheads in insets), which implicated massive binding of protein complexes to the I-bands. Scale bar, 1 µm. (b) Expression levels of the chaperones HSP27, aB-crystallin (aBC) and HSP90 in diseased human muscles by Western blotting, indexed to the mean levels in human CTRL muscles. Data on graphs are means±SEM (n=3 per group). ***p: <0.001 in Bonferroni adjusted t-test.

## Slide 2
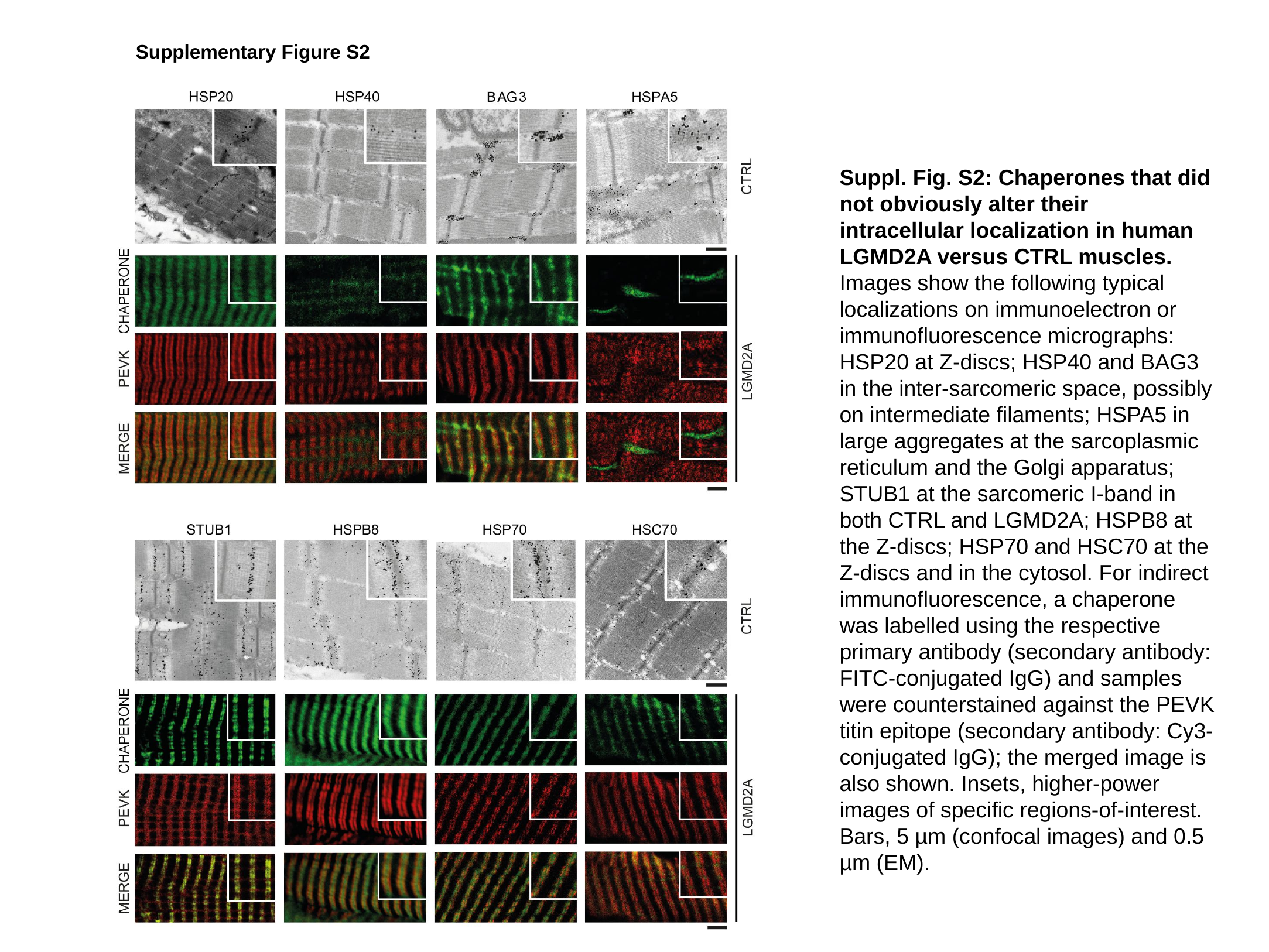

Supplementary Figure S2
Suppl. Fig. S2: Chaperones that did not obviously alter their intracellular localization in human LGMD2A versus CTRL muscles. Images show the following typical localizations on immunoelectron or immunofluorescence micrographs: HSP20 at Z-discs; HSP40 and BAG3 in the inter-sarcomeric space, possibly on intermediate filaments; HSPA5 in large aggregates at the sarcoplasmic reticulum and the Golgi apparatus; STUB1 at the sarcomeric I-band in both CTRL and LGMD2A; HSPB8 at the Z-discs; HSP70 and HSC70 at the Z-discs and in the cytosol. For indirect immunofluorescence, a chaperone was labelled using the respective primary antibody (secondary antibody: FITC-conjugated IgG) and samples were counterstained against the PEVK titin epitope (secondary antibody: Cy3-conjugated IgG); the merged image is also shown. Insets, higher-power images of specific regions-of-interest. Bars, 5 µm (confocal images) and 0.5 µm (EM).

## Slide 3
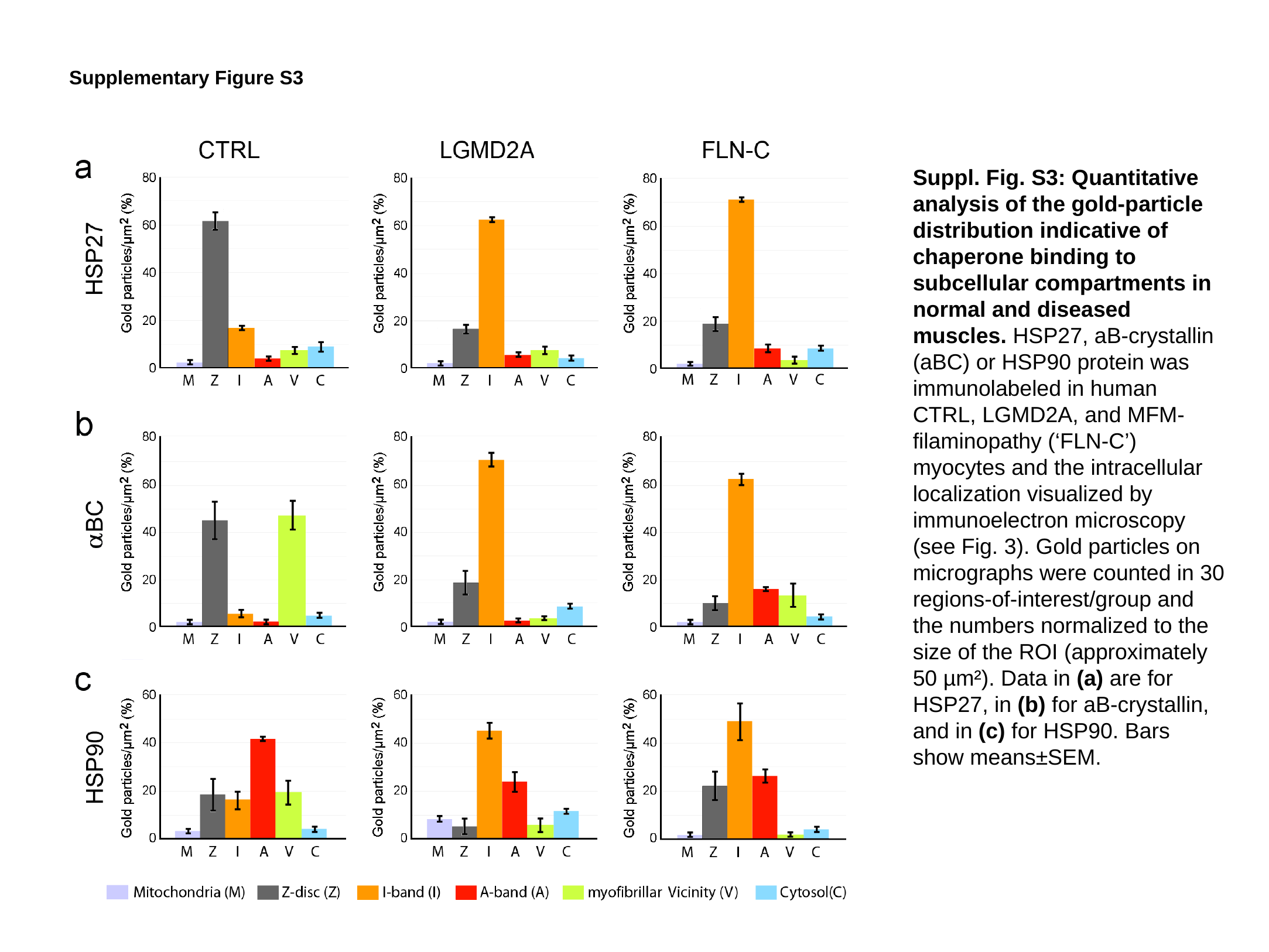

Supplementary Figure S3
Suppl. Fig. S3: Quantitative analysis of the gold-particle distribution indicative of chaperone binding to subcellular compartments in normal and diseased muscles. HSP27, aB-crystallin (aBC) or HSP90 protein was immunolabeled in human CTRL, LGMD2A, and MFM-filaminopathy (‘FLN-C’) myocytes and the intracellular localization visualized by immunoelectron microscopy (see Fig. 3). Gold particles on micrographs were counted in 30 regions-of-interest/group and the numbers normalized to the size of the ROI (approximately 50 µm²). Data in (a) are for HSP27, in (b) for aB-crystallin, and in (c) for HSP90. Bars show means±SEM.

## Slide 4
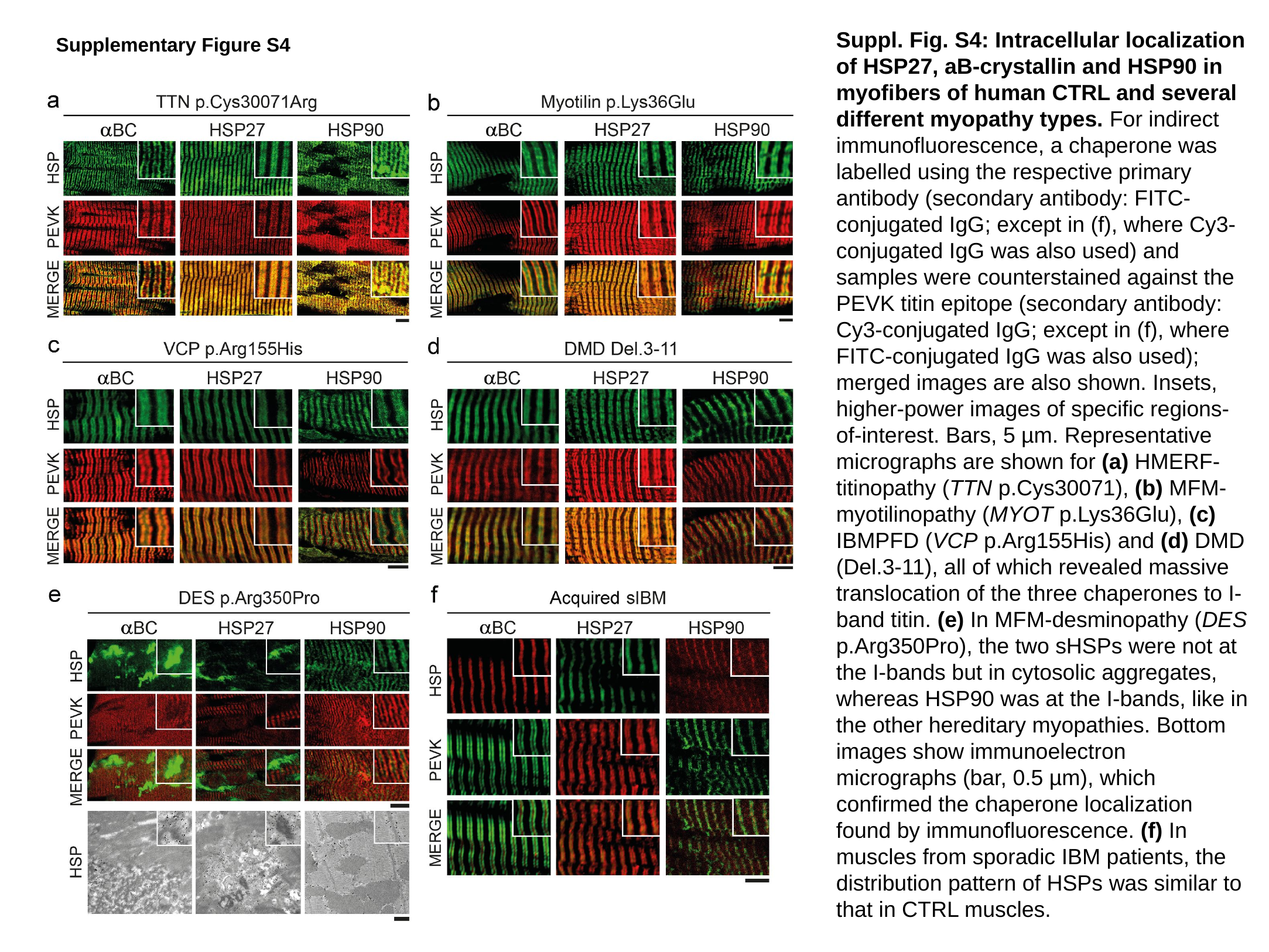

Suppl. Fig. S4: Intracellular localization of HSP27, aB-crystallin and HSP90 in myofibers of human CTRL and several different myopathy types. For indirect immunofluorescence, a chaperone was labelled using the respective primary antibody (secondary antibody: FITC-conjugated IgG; except in (f), where Cy3-conjugated IgG was also used) and samples were counterstained against the PEVK titin epitope (secondary antibody: Cy3-conjugated IgG; except in (f), where FITC-conjugated IgG was also used); merged images are also shown. Insets, higher-power images of specific regions-of-interest. Bars, 5 µm. Representative micrographs are shown for (a) HMERF-titinopathy (TTN p.Cys30071), (b) MFM-myotilinopathy (MYOT p.Lys36Glu), (c) IBMPFD (VCP p.Arg155His) and (d) DMD (Del.3-11), all of which revealed massive translocation of the three chaperones to I-band titin. (e) In MFM-desminopathy (DES p.Arg350Pro), the two sHSPs were not at the I-bands but in cytosolic aggregates, whereas HSP90 was at the I-bands, like in the other hereditary myopathies. Bottom images show immunoelectron micrographs (bar, 0.5 µm), which confirmed the chaperone localization found by immunofluorescence. (f) In muscles from sporadic IBM patients, the distribution pattern of HSPs was similar to that in CTRL muscles.
Supplementary Figure S4

## Slide 5
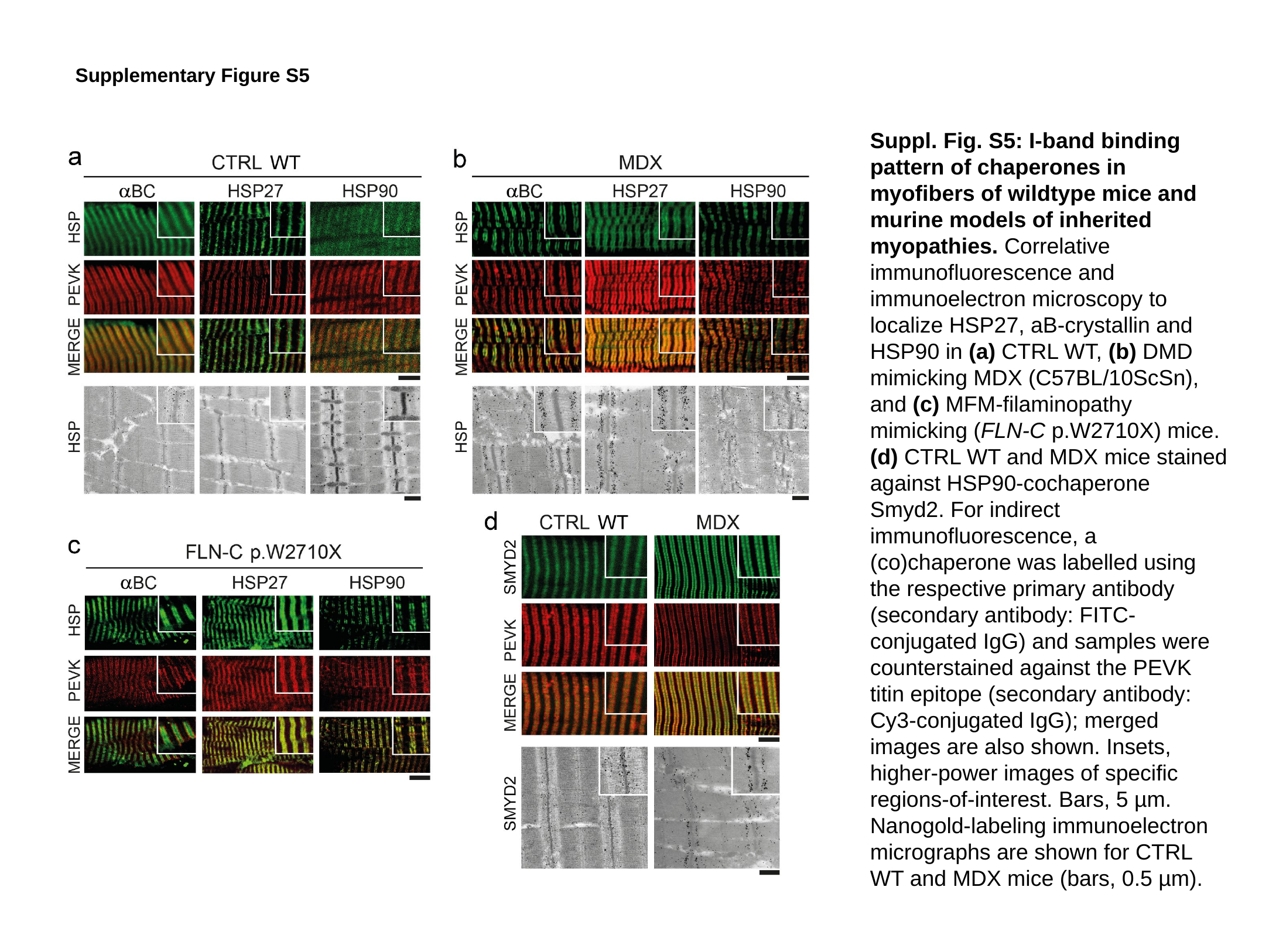

Supplementary Figure S5
Suppl. Fig. S5: I-band binding pattern of chaperones in myofibers of wildtype mice and murine models of inherited myopathies. Correlative immunofluorescence and immunoelectron microscopy to localize HSP27, aB-crystallin and HSP90 in (a) CTRL WT, (b) DMD mimicking MDX (C57BL/10ScSn), and (c) MFM-filaminopathy mimicking (FLN-C p.W2710X) mice. (d) CTRL WT and MDX mice stained against HSP90-cochaperone Smyd2. For indirect immunofluorescence, a (co)chaperone was labelled using the respective primary antibody (secondary antibody: FITC-conjugated IgG) and samples were counterstained against the PEVK titin epitope (secondary antibody: Cy3-conjugated IgG); merged images are also shown. Insets, higher-power images of specific regions-of-interest. Bars, 5 µm. Nanogold-labeling immunoelectron micrographs are shown for CTRL WT and MDX mice (bars, 0.5 µm).
